# Assessing Silicon-Mediated Growth Performances in Contrasting Rice Cultivars under Salt Stress

**DOI:** 10.3390/plants11141831

**Published:** 2022-07-13

**Authors:** Uzzal Somaddar, Hridoy Chandra Dey, Sarah Khanam Mim, Uttam Kumer Sarker, Md. Romij Uddin, Nasar Uddin Ahmed, Mohammad Golam Mostofa, Gopal Saha

**Affiliations:** 1 Department of Agronomy, Patuakhali Science and Technology University, Dumki, Patuakhali 8602, Bangladesh; u.somaddar@gmail.com; 2 Faculty of Agriculture, Patuakhali Science and Technology University, Dumki, Patuakhali 8602, Bangladesh; hridoydey879@gmail.com (H.C.D.); sarahkhanammim@gmail.com (S.K.M.); 3 Department of Agronomy, Bangladesh Agricultural University, Mymensingh 2202, Bangladesh; uttam@bau.edu.bd (U.K.S.); romijagron@bau.edu.bd (M.R.U.); 4 Department of Genetics and Plant Breeding, Patuakhali Science and Technology University, Dumki, Patuakhali 8602, Bangladesh; nuahmed@pstu.ac.bd; 5 Institute of Genomics for Crop Abiotic Stress Tolerance, Department of Plant and Soil Science, Texas Tech University, Lubbock, TX 79409, USA; mmostofa@ttu.edu

**Keywords:** electrolyte leakage, proline, rice, salinity, silicon

## Abstract

Silicon (Si) application has great potential to improve salt tolerance in a variety of crop plants. However, it is unclear how Si influences the responses of contrasting rice cultivars when exposed to excessive salt. Here, we investigated the functions of Si in alleviating the negative effects of salt stress on two contrasting rice cultivars, namely BRRI dhan48 (salt-sensitive) and Binadhan-10 (salt-tolerant). Rice seedlings was pre-treated with three doses of Si (as silicic acid; 0, 1 and 2 mM) for 14 days at one-day interval before being exposed to salt stress (10 dSm^−1^) in a sustained water bath system. The results demonstrated that the seedlings of BRRI dhan48 and Binadhan-10, respectively exhibited substantial reductions in shoot height (16 and 9%), shoot fresh weight (64 and 43%) and shoot dry weight (50 and 39%) under salinity. Intriguingly, BRRI dhan48 pre-treated with 1 and 2 mM Si, respectively, showed a higher increase in shoot height (SH) (by 25.90 and 26.08%) as compared with Binadhan-10 (by 3 and 8%) under salt stress compared with their respective controls. Data revealed that a comparatively higher improvement in the growth performances of the salt-induced Si pre-treated BRRI dhan48 than that of Binadhan-10. For example, 1 and 2 mM of Si treatments significantly attributed to elevated leaf relative water content (RWC) (13 and 22%), proline (138 and 165%), chlorophyll *a* (42 and 44%), chlorophyll *b* (91 and 72%), total chlorophyll (58 and 53%) and carotenoids (33 and 29%), and recovery in the reductions of electrolyte leakage (13 and 21%), malondialdehyde content (23 and 30%) and shoot Na^+^/K^+^ ratio (22 and 52%) in BRRI dhan48 compared with Si-untreated control plants under salt stress. In addition, we found salt-tolerant Binadhan-10 also had enhanced RWC (9 and 19%), proline (12 and 26%) with pre-treatment with 1 and 2 mM of Si, respectively, under salt stress, while no significant differences were noticed in the case of photosynthetic pigments and Na^+^/K^+^ ratio. Our results showed that Si supplementation potentiated higher salt-tolerance ability in the salt-sensitive BRRI dhan48 as compared with salt-tolerant Binadhan-10. Thus, Si application could be highly beneficial in the growth recovery of the salinity-affected salt-sensitive high yielding rice cultivars in the saline-prone areas.

## 1. Introduction

Rice (*Oryza sativa*) is the most important food crop and more than 50% of the world’s population consumes it as a staple food [1]. Rice plants at seedling stage are sensitive to salinity, which limits crop production and creates enormous economic loss globally [2]. The global climate change has exacerbated this problem worldwide. Thus, improving rice resilience to salt stress is a crucial issue in overcoming the failing food production system and fulfilling the world’s ever-increasing food demands [3]. Plants growing under salinity experience two types of primary stresses, including osmotic stress and ionic stress [4]. Both of these stresses can further generate a secondary stress, namely oxidative stress in plant tissues [5]. Salt stress reduces the physiological functions of the plants like photosynthesis and osmotic adjustment [6]. The ionic and osmotic salinity components both result in the accumulation of reactive oxygen species (ROS) and membrane peroxidation [4,7]. Thus, high salinity decreases the agricultural productivity through reductions in plants’ photosynthetic capacity, water use efficiency, and total fresh and dry biomass [8].

Silicon (Si) has been regarded as a beneficial element for plant growth. However, plant species cannot uptake Si equally, which is one of the main factors for differential Si accumulation in the above ground parts of plants [9,10]. For example, Si concentrations in plants vary between 0.1 and 10% on a dry weight basis [11]. Rice is normally an Si accumulator, and it can accumulate up to 10% Si, which is higher than the concentrations of essential macronutrients like nitrogen, phosphorus, and potassium that could be accumulated by rice [10]. Plants’ tolerance to both biotic and abiotic stress is known to improve with silicon accumulation [12,13]. Exogenous Si treatment has been a new environmentally acceptable technique to improve salt stress response in plants [14]. Si application in rice has been reported to improve the salinity tolerance through reducing the ionic and osmotic constituents [15]. Several reports revealed that Si treatment potentiate salt tolerance in a variety of crops, including rice, [16], wheat (*Triticum aestivum*) [17,18], barley (*Hordeum vulgare*) [19,20,21], sugarcane (*Saccharum officinarum*) [22,23], soybean (*Glycine max*) [24], and zucchini (*Cucurbita pepo*) [25]. Thus, Si supplementation as inclusion of Si-fertilizer or any innovation regarding this in increasing the crop productivity in the saline-prone areas would be an impending solution due to its cost-effectiveness and eco-friendliness [26,27].

Salinity causes the accumulation of suitable soluble compounds or osmolytes (e.g., proline, glycine betaine, mannitol, sugar, N-containing compounds for example, polyamines, amino acids, etc.); those at high concentrations help to adapt salt-induced negative impacts in plants [28,29]. For instance, proline accretion in tissues under salt stress appears in many plant species including rice [30,31]. Proline, along with other amino acids like arginine, cysteine and methionine, helps plants to adapt in salt stress by decreasing NaCl-induced K^+^ efflux and maintaining K^+^ homeostasis [32]. Thus, monitoring of proline content under salt-induced conditions could be an important indicator to assess the Si-mediated salt tolerance mechanism in plants. Based on the high potential roles of Si, the current study aimed to investigate the Si-mediated salt tolerance mechanisms in the contrasting (salt -tolerant and -sensitive) rice genotypes based on comparative morpho-physiological and biochemical indices like growth performances, and their leaf chlorophyll content, relative water content (RWC), lipid peroxidation product (malondialdehyde, MDA) and accumulation of Na^+^, K^+^ and proline.

## 2. Results

### 2.1. Effect of Silicon on Growth Performances of Rice Seedlings under Salinity

**Figure 1:**
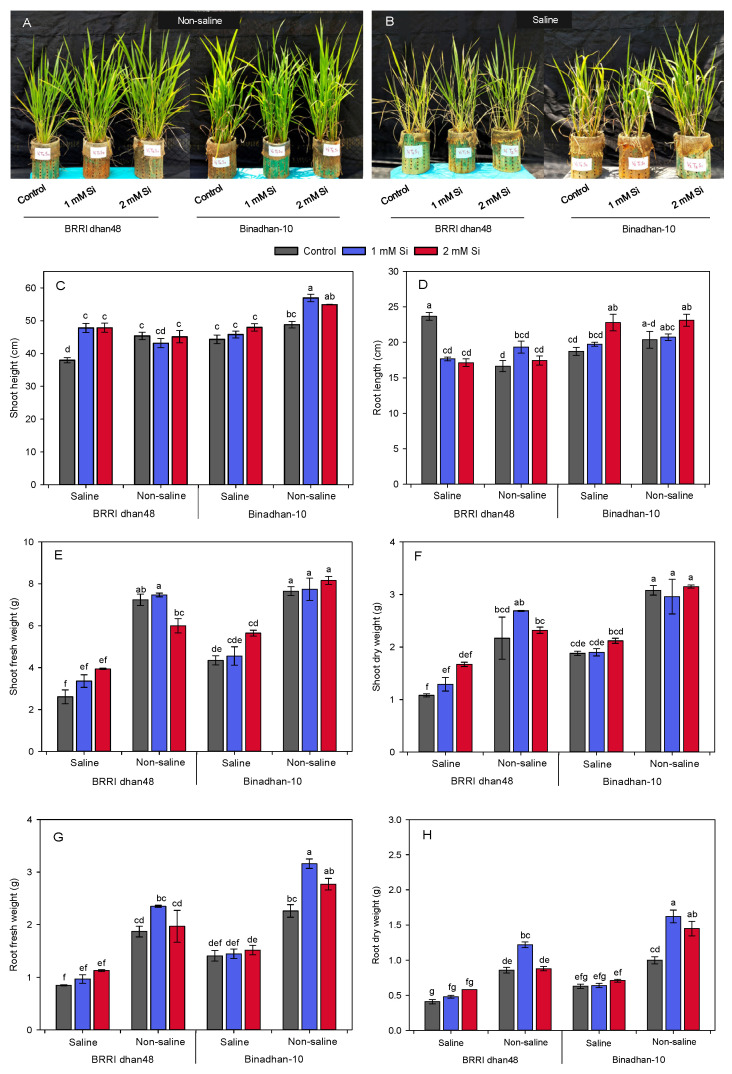
Effect of Si supplementation (1 and 2 mM silicic acid) on rice plants subjected to salt stress (10 dSm^−1^ NaCl). (**A**,**B**) Showing comparative phenotypes of BRRI dhan48 (salt-sensitive) and Binadhan-10 (salt-tolerant) with or without Si pre-treatment under salinity, (**C**) shoot length, (**D**) root length, (**E**) shoot fresh weight, (**F**) shoot dry weight, (**G**) root fresh weight and (**H**) root dry weight were recorded after six days of recovery from salt tress. Different letters above the bars indicate significant differences among the treatments following Tukey’s HSD test.

Pre-treatment of Si (in the form of silicic acid) significantly modulated several growth attributes of rice seedlings under salt stress (Figure 1). We observed a notable improvement on the salt-induced phenotype of both the salt-sensitive and-tolerant rice cultivars in response to Si pre-treatment as compared with their control (i.e., Si untreated conditions) (Figure 1A,B). We recorded a significant reduction in shoot height in both BRRI dhan48 and Binadhan-10 (16 and 9%, respectively) after being exposed to salt stress compared with unstressed conditions (Figure 1C). Interestingly, pre-treatment with 1 and 2 mM Si contributed to an increase in the shoot height of BRRI dhan48, respectively, by 25.90 and 26.08% (*p* < 0.01), while no such significant differences were observed in the case of salt-tolerant Binadhan-10 under salt stress. In addition, root length of salt-induced BRRI dhan48 was found to be reduced by 25 and 28%, respectively, after 1 and 2 mM of Si supplementation. In contrast, Binadhan-10 demonstrated a significant increase in root length (22%) compared with control, while pre-treated with 2 mM Si under saline conditions (Figure 1D).

In addition, shoot fresh and dry weights have been recorded to decrease remarkably in both BRRI dhan48 and Binadhan-10 after their salt exposure (Figure 1E,F). The addition of 1 and 2 mM of Si have non-significantly improved shoot fresh weight, respectively, by 29 and 51%, and shoot dry weight, respectively by 19 and 54% in salt-induced BRRI dhan48 compared with their Si-untreated control (Figure 1E,F). Tolerant cultivar Binadhan-10 also did not show significant improvement in shoot fresh and shoot dry weights in response to Si pre-treatment (Figure 1E,F). In addition, in the Si-untreated salt stress condition, root fresh and dry weight in salt-sensitive BRRI dhan48 have been reduced by 55 and 52%, respectively, while the salt-tolerant Binadhan-10 incurred reductions of 38 and 36%, respectively. In our study, pre-treatment with 1 and 2 mM Si could not significantly contribute to improving root fresh and root dry weights (Figure 1G,H). However, while considering the interaction effect between salinity levels and Si treatment, we observed significant relationships in the case of shoot and root fresh weight, and root dry weight (Table S1).

### 2.2. Effect of Silicon on Different Physiological and Biochemical Parameters

**Figure 2:**
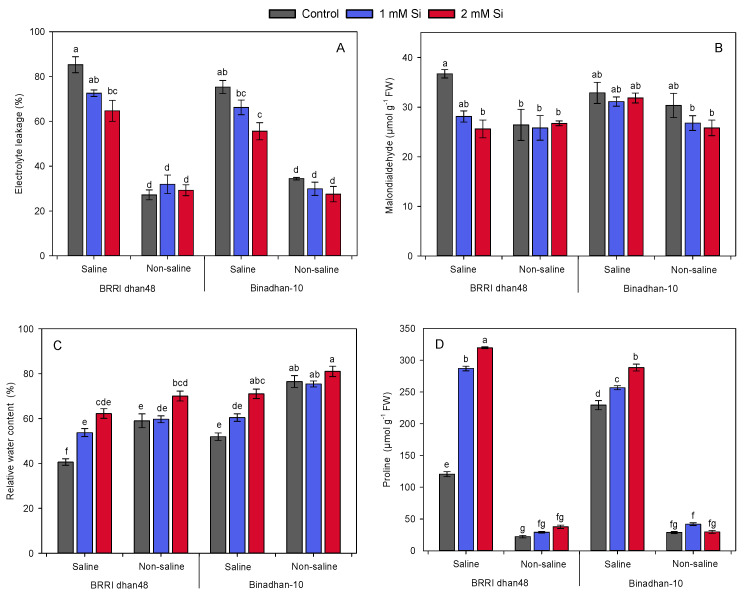
Effect of exogenous Si application (1 and 2 mM silicic acid) on different physiological and biochemical parameters in rice plants subjected to salt stress (10 dSm^−1^). (**A**) Electrolyte leakage, (**B**) malondialdehyde content, (**C**) relative water content and (**D**) proline content were recorded after six days of recovery from salt stress. Different letters above the bars indicate significant differences among the treatments following Tukey’s HSD test.

Salt stress enhanced electrolyte leakage (%EL) in both sensitive (BRRI dhan48) and tolerant (Binadhan-10) cultivars by 58 and 41%, compared with those under non-saline conditions. EL has significantly decreased in the Si-pre-treated contrasting cultivars under salt-stress (Figure 2A). After supplementation with 1 and 2 mM Si, the EL in both the salt-induced BRRI dhan48 and Binadhan-10 reduced, respectively, by 13 & 22% and 9 & 20%, compared with their respective controls i.e., in Si-untreated saline conditions (Figure 2A). In addition, salt stress induced the highest amount of MDA generation in the leaves of BRRI dhan48 (36.73 µmol g^−1^ FW), compared with that in the cases of BRRI dhan48 (26.43 µmol g^−1^ FW) and Binadhan-10 (30.35 µmol g^−1^ FW) under non-saline conditions (Figure 2B). Pre-treatment with both 1 and 2 mM of Si led to significant reductions in MDA levels (23 and 30%, respectively) in the salt-stressed BRRI dhan48, while no significant variations were observed in the cases of salt-induced Binadhan-10 (Figure 2B).

Pre-treatment with 1 and 2 mM Si under salt-stress significantly increased the RWC in both BRRI dhan48 (respectively by 13 and 22%) and Binadhan-10 (respectively by 9 and 19%), compared with their respective controls (Si-untreated plants) (Figure 2C).

In addition, salt stress induced a higher accumulation of proline in both of the rice cultivars (Figure 2D). Pre-treatment with 1 and 2 mM of Si caused a further increase in leaf proline levels under salt stress. Meanwhile, comparing the proline content between two rice cultivars, higher proline accumulation was recorded in the salt-tolerant Binadhan-10 than salt-sensitive BRRI dhan48 in Si-untreated conditions. However, after pre-treatment with Si, the proline content became higher in the BRRI dhan48 than in Binadhan-10 under salt stress (Figure 2D). For instance, supplementation of 1 and 2 mM of Si in the salt-sensitive BRRI dhan48 upregulated the leaf proline content, respectively, by 138 and 165%, compared with those under Si-untreated condition, whereas the tolerant cultivar Binahan-10 pre-treated with 1 and 2 mM Si, respectively, only had 12 and 26% increased leaf proline compared with Si-untreated control (Figure 2D).

### 2.3. Effect of Silicon on Photosynthetic Pigments

**Figure 3:**
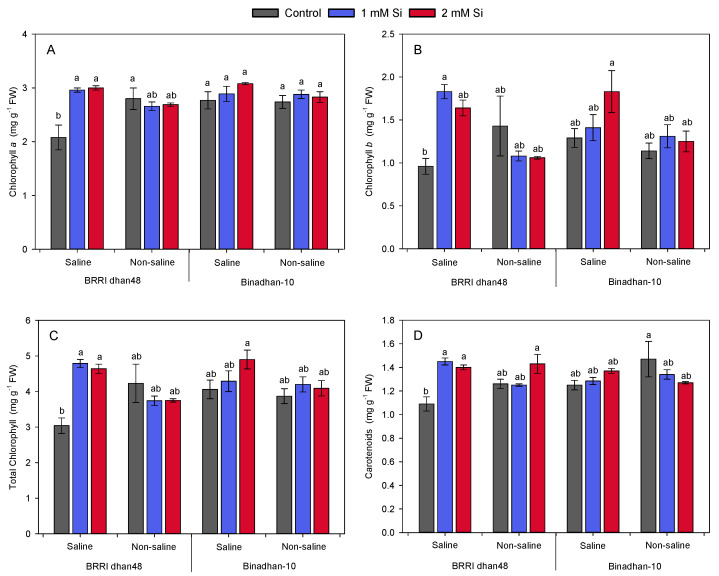
Effect of exogenous Si (1 and 2 mM silicic acid) on rice plants subjected to salt stress (10 dSm^−1^). (**A**) Chlorophyll *a*, (**B**) chlorophyll *b*, (**C**) total chlorophylls and (**D**) carotenoids were recorded in the leaves of two contrasting rice cultivars after six days of recovery from salt stress. Different letters above the bars indicate significant differences among the treatments following Tukey’s HSD test.

Without Si pre-treatment, salt stress significantly reduced the levels of photosynthetic pigments (chlorophyll *a*, chlorophyll *b*, total chlorophyll and carotenoids) in the salt-sensitive BRRI dhan48. Meanwhile, the salt-tolerant cultivar Binadhan-10 maintained remarkable stability between saline and non-saline conditions (Figure 3A,D). Si pre-treatment alleviated the salt stress-induced chlorophyll degradation in BRRI dhan48. Neither Si nor salt treatments caused significant changes in pigment composition in the salt-tolerant Binadhan-10 genotype. Notably, in response to 1 mM Si pre-treatment, the concentration of chlorophyll *a*, chlorophyll *b*, total chlorophyll and carotenoids were increased by 42, 91, 58 and 33%, respectively, in the salt-stressed BRRI dhan48 compared with Si-untreated control plants (Figure 3A,D).

### 2.4. Effect of Silicon on the Na^+^ and K^+^ Content of Rice Shoot

**Figure 4:**
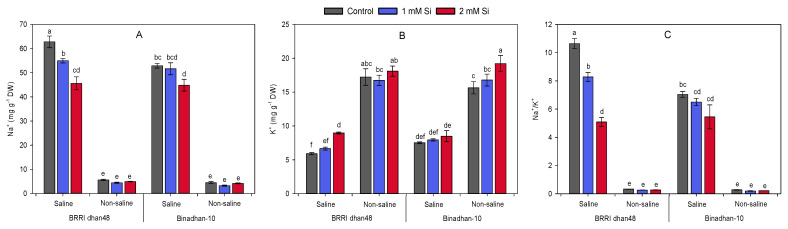
Effect of Si pre-treatment (1 and 2 mM silicic acid) on (**A**) Na^+^, (**B**) K^+^ content and (**C**) Na^+^/K^+^ ratio in the shoots of two contrasting rice cultivars subjected to salt stress (10 dSm^−1^). Different letters above the bars indicate significant differences among the treatments following Tukey’s HSD test.

Si pre-treatment caused a significant reduction in Na^+^ accumulation and improvement in K^+^ concentration to maintain Na^+^/K^+^ balance in the shoots of both rice cultivars under salt stress (Figure 4A,C). Under salt stress, the shoots of BRRI dhan48 and Binadhan-10 accumulated remarkably higher Na^+^, which are 62.78 and 54.96 mg g^−1^ DW, respectively, without Si pre-treatment, while pre-treatment with 1 and 2 mM Si reduced the Na^+^ accumulation in both BRRI dhan48 (12 and 27%, respectively) and Binadhan-10 (2 and 15%, respectively) compared with their respective control plants under Si-untreated conditions (Figure 4A). Meanwhile, no significant variation was observed in response to exogenous Si application under a non-saline environment (Figure 4A). Our data also revealed that 2 mM Si treatment could contribute to preventing the K^+^ loss of shoot under salt stress conditions in both BRRI dhan48 and Binadhan-10. However, Si-treatment also increased K^+^ concentration under non-saline condition, particularly in Binadhan-10 (Figure 4B). As a whole, salt stress elevated the Na^+^/K^+^ ratio in the Si untreated conditions, which have subsequently been reduced significantly with varying levels of Si application (1 and 2 mM) in both BRRI dhan48 (22 and 52%, respectively) and Binadhan-10 (8 and 23%, respectively). Notably, no remarkable effect of Si was observed in the Na^+^/K^+^ ratio under non-saline conditions (Figure 4C).

### 2.5. Correlation Analysis among Different Growth and Physiological Parameters

**Figure 5:**
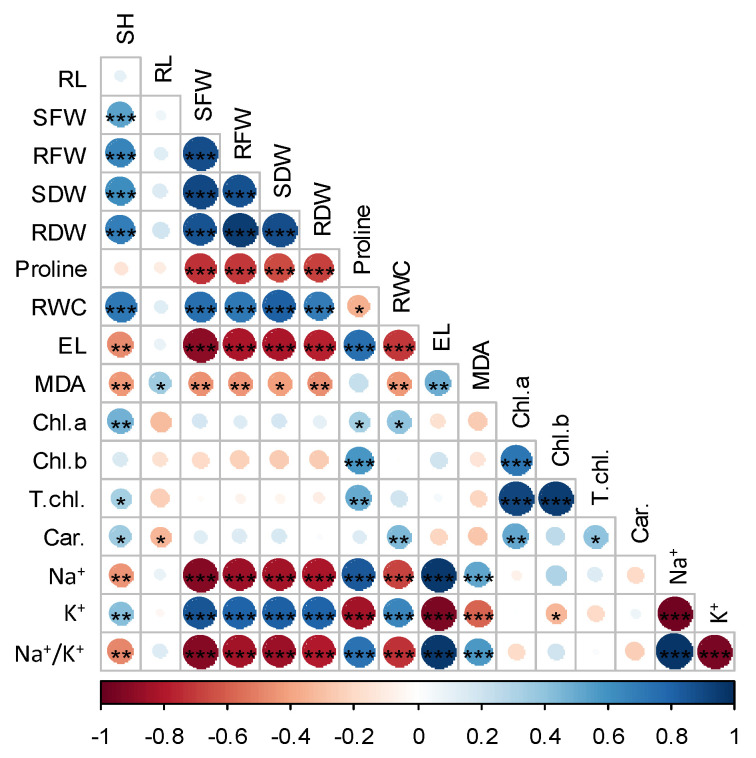
Pearson correlation matrix for different growth and physiological attributes of Si supplemented two rice cultivars under salt stress. SH, shoot height; RL, root length; SFW, shoot fresh weight; RFW, root fresh weight; SDW, shoot dry weight; RDW, root dry weight; RWC, relative water content; EL, electrolyte leakage; MDA, malodialdehyde; Chl, chlorophyll; T. chl, total chlorophyll; Car, carotenoid; Na^+^, sodium and K^+^, potassium. *, ** and *** indicating significant correlation at *p* < 0.05, *p* < 0.01 and *p* < 0.001 levels, respectively. Heatmap shows negative and positive correlations.

We performed a correlation analysis to identify how different growth and physiological characteristics were interrelated in the two contrasting rice seedlings under salinity (Figure 5). We observed that proline content had a strong negative correlation with shoot-root fresh and dry weight, and K^+^, while having a positive correlation with EL, Chl *a*, Chl *b*, tolal Chl, Na^+^ and Na^+^/K^+^ ratio (*p* < 0.001 and *p* < 0.01). An overall significant positive correlation was evident between RWC and growth characters, Chl *a* and Car., but negatively correlated with EL, MDA and Na^+^/K^+^ ratio. We also found a negative correlation in case of EL, RWC and different growth parameters, while it was positively correlated with MDA and Na^+^/K^+^ ratio. The overall findings indicated the salt-induced negative effects in rice, which might be inhibited by the stimulatory growth effects attributed by Si pre-treatment (Figure 5).

### 2.6. Principal Component Analysis

**Figure 6:**
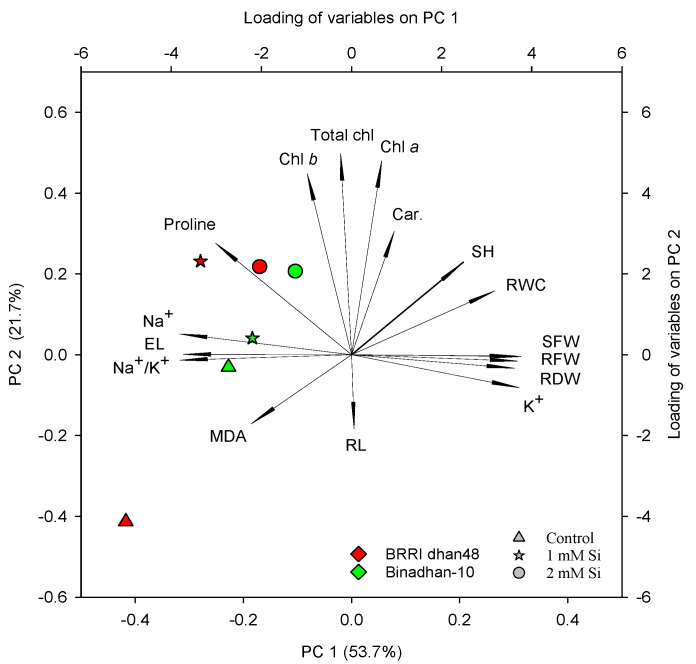
Bi-plot of principal component analysis (PCA) showing the first two principal components (PC 1 and PC 2). For PCA, data on growth and physiological attributes of both BRRI dhan48 and Binadhan-10 rice seedlings under salt stress. Different shapes in the bottom right denote three Si treatments, and red and green colors, respectively, indicating two rice cultivars like BRRI dhan48 and Binadhan-10. SH, shoot height; RL, root length; SFW, shoot fresh weight; RFW, root fresh weight; SDW, shoot dry weight; RDW, root dry weight; Chl *a*, chlorophyll *a*; Chl *b*, chlorophyll *b*; Total chl, total chlorophyll; Car., total carotenoids; RWC, relative water content; EL, electrolyte leakage and MDA, malondialdehyde.

We used principal component analysis to determine the parameters that best describe salt tolerance by identifying the key variables that explain the pattern of correlations within the detected salinity stress component characteristics (Figure 6). The bi-plot of the first two principal components (PCs) and the loading of variables are presented in Figure 6. The first and second PC explained, respectively, 53.7% and 21.7% of the variation in rice seedlings. The proline content and photosynthetic pigments (Chlorophyll *a*, *b* and carotenoids), shoot height, RWC, and shoot and root fresh weights are positively associated under salt stress with different Si treatments (Control, 1 and 2 mM Si). In addition, root length, MDA, EL and Na^+^/K^+^ showed a negative correlation with photosynthetic pigments, RWC and different growth characters under salinity stress, indicating the positive role of Si in alleviation of salt stress in rice seedlings (Figure 6). On the other hand, shoot height, RWC, and fresh and dry weight of shoot were positively correlated with each other; however, root length had a negative correlation with shoot height and RWC under non-saline condition (Figure 6).

## 3. Discussion

Salinity is one of the most serious environmental threats that is affecting agricultural production, resulting in higher economic loss globally [33]. In our present study, we investigated the Si-mediated growth performances in two contrasting rice genotypes, namely BRRI dhan48 (sensitive) and Binadhan-10 (tolerant) under salt stress. Important growth parameters like shoot height and root length have been significantly hampered under salt stress (Figure 1). Interestingly, the application of Si reduced the salt-induced negative impacts through improvement in shoot height of the salt-sensitive BRRI dhan48, while the control plants of salt-tolerant group (Binadhan-10) showed more stability with the Si pre-treatment under salt stress. Si addition decreased the root length in salt-induced BRRI dhan48 but increased in the Binadhan-10 compared with their respective controls (Figure 1D). Several reports also revealed that shoot height, shoot fresh and dry weight increased with Si supplement, thus reducing the salinity effect in barley and rice [27,34,35,36].

Under stress conditions, EL and MDA are predicted to be important indicators of cellular damage [37]. EL value reflects the extent of membrane damage and cellular integrity, while MDA represents the oxidative burst induced by salinity or any other stresses [38]. Salinity stress tends to increase cellular injury by enhancing EL [13]. Our findings demonstrated that salinity stress causes higher EL and MDA, particularly in the salt-sensitive BRRI dhan48, probably due to cellular oxidative damage. However, Si pre-treatment reduced salt-induced damage, which is indicated by the lower EL and MDA (Figure 2A,B). For instance, pre-treatment with Si (1 and 2 mM) caused significant reductions in MDA levels (23 and 30%, respectively) in the salt-stressed BRRI dhan48, while no significant variations in MDA were observed in the salt-induced Binadhan-10 (Figure 2B). Our results are consistent with the findings of Yan et al. [15] and Abdelaal et al. [39], where they reported that Si application reduced EL and MDA in rice and sweet pepper under salinity. Furthermore, we observed that leaf RWC and proline status were increased in the salt-induced rice plants when treated with 1 and 2 mM of Si (Figure 2C,D). Studies revealed that Si improves total water balance in plants through deposition in the leaf surface, which play important roles in reducing water loss through a slower rate of transpiration [40]. Abdelaal et al. [39] also observed that leaf RWC and proline content were also increased in the salt-induced plants after foliar application with Si in sweet pepper. Further results also showed that Si application in the salt-stressed rice and cucumber seedlings improved leaf transpiration rate possibly by regulating the water uptake process [16,41]. In addition, plants under salt stress have been reported to activate salt-mediated osmotic stress pathways to synthesize different compatible osmolytes like proline, betaine and soluble sugars, thus enhancing the performance of osmoregulatory functions in plants [42,43]. In sorghum and wheat, Si application under salt stress significantly altered the levels of leaf soluble sugar and proline compared with controls [44,45]. Our data in this regard also corroborate with these findings, where we found that Si pre-treatment caused higher improvement in the leaf proline levels in salt-sensitive BRRI dhan48 than in salt-tolerant Binadhan-10 under salt stress (Figure 2D).

In addition, several studies highlighted that Si supplementation under salt-stress has been highly effective in mitigating salinity-induced negative impacts mainly by reducing the Na^+^ accumulation in the roots and/or shoots of plants [46]. For example, in barley, Si-mediated recovery of salinity stress has mainly occurred by restricting Na^+^ entry into tissues [47]. Variability in Na^+^ accumulation in the root and shoot tissues has also been reported by several researchers [18,48]. For instance, in alfalfa (*Medicago sativa*), Si addition significantly reduced the Na^+^ concentration in root, while no such impact was observed in the case of shoots [48]. In addition, in wheat, Si supplementation simultaneously lowered the Na^+^ toxicity in both roots and shoots [18]. In connection to our study, Si application has also been reported to alleviate salt stress in rice. Here, we observed that varying levels of Si pre-treatments have significantly reduced shoot Na^+^ level in both salt-sensitive and salt-tolerant rice genotypes under salinity (Figure 4A). Gong et al. [16] found that rice plants supplemented with Si under salinity did not show any change in root Na^+^ levels compared with control, while the upward transport of Na^+^ had reduced through the apoplastic pathway and lowered the shoot Na^+^ concentration. Reports revealed that, after Si application in rice, thicker levels of casparian bands both in the exodermis and endodermis were formed, which might play partial roles in restricting Na^+^ from entering the symplastic system or transpiration stream [49,50]. Si supplementation has also been reported to augment the cellular K^+^ content in plants under salinity [13]. We found that 2 mM Si pre-treatment contributed to prevent the K^+^ loss of shoot in both BRRI dhan48 and Binadhan-10 under salt stress conditions. Moreover, Si-treatment also increased K^+^ concentration under non-saline condition, particularly in Binadhan-10 (Figure 4B). Thus, plants supplemented with Si under salinity maintain a favorable Na^+^/K^+^ ratio even in higher concentrations of salt stress and improve salinity tolerance [51,52].

Our study also indicated that salinity stress considerably reduced photosynthetic pigments (chlorophyll *a*, chlorophyll *b*, total chlorophyll, and carotenoids) in the salt-sensitive BRRI dhan48 compared with unstressed plants (Figure 3). Pre-treatment with 1 and 2 mM Si significantly improved photosynthetic pigment levels in the salt-sensitive BRRI dhan48 under salt stress. However, Binadhan-10 maintained the highest and stable photosynthetic pigment composition in all of the treatment conditions (Figure 3A,D). Likewise, Si application played positive roles in recovery of chlorophyll *a* and *b* in salt-induced sweet pepper plants [39]. Ibrahim et al. [53] and Rios et al. [54] also reported about the increase in chlorophyll *a*, chlorophyll *b*, and carotenoids in wheat leaves after Si application under salt stress. Thus, results presented in our study also signify the positive roles of Si in improving seedling salinity tolerance in the salt-sensitive BRRI dhan48, which might be attributed by increased chlorophyll synthesis and associated morpho-physiological growth parameters under salinity.

## 4. Materials and Methods

### 4.1. Plant Materials and Experimental Procedure

Two contrasting rice genotypes Binadhan-10 (salt-tolerant) and BRRI dhan48 (salt-sensitive) were collected from the Bangladesh Institute of Nuclear Agriculture (BINA) and Bangladesh Rice Research Institute (BRRI), respectively. The experiment was conducted in the field laboratory (net house) and the laboratory analyses were carried out at the stress agronomy laboratory of Patuakhali Science and Technology University, following the method as described by Gregorio et al. [55]. Briefly, four to five pre-germinated seeds were sown in porous metal pots (50 cm × 30 cm × 20 cm) with 3 kg of puddled rice field soil. To prepare the growth medium, per kg rice field soil was treated with 50 mg urea, 25 mg tripple superphosphate and 25 mg muriate of potash. Poorly established seedlings were eliminated at seven days of seeding age, and, eventually, three seedlings per pot were kept for treatment application. The metal pots were placed in a stainless steel water bath to maintain the proper water level for the developing seedlings. The water level was checked on a regular basis, and pest and disease control measures were taken as per necessity.

Rice seedlings were pre-treated with Si {in the form of silicic acid (SiO_2_.H_2_O)} @ 1 and 2 mM. Si pre-treatment was done in the pots of 7-day-old rice seedlings for 14 days on alternate days. To compare the Si effect, only tap water was used as control following the similar Si-treatment procedure. One week after the Si pre-treatment salt stress (10 dSm^−1^) was imposed on the 21-day-old rice seedlings through addition of 5.2 g/L of NaCl in the water bath for 15 days. Following the same growing conditions, a non-saline culture condition was also maintained using tube well water at the base value. The pH of both the saline and non-saline culture medium was maintained between 5.0–5.1 using 0.01 N of HCl or 0.1 N NaOH.

Thus, a three-factor experiment was set up in a completely randomized design (CRD). Here, two rice varieties, namely Binadhan-10 (salt-tolerant) and BRRI dhan48 (salt-sensitive) as factor A, salinity levels non-saline and saline (10 dsm^−1^) as factor B, and three treatments of Si (control-tube well water, 1 mM and 2 mM Si) as factor C were used in this experiment. The experiment was replicated three times, and all the morpho-physiological and biochemical assays were performed using three biological replicates.

### 4.2. Estimation of Shoot Height, Fresh and Dry Weight of Shoot-Root

Shoot height was recorded from 42-day-old rice seedlings using a meter scale. Three plants from each container were sampled and separated into the shoots and roots. The shoot and root were wiped with tissue paper to avoid excess moisture, and their fresh weight was taken. The samples were then kept in an oven at 70 °C for 72 h, and dry weight was taken.

### 4.3. Photosynthetic Pigments Analysis (Chlorophyll a, b, Total Chlorophyll and Carotenoid)

Photosynthetic pigments were analyzed following the modified method of Arnon [56] and Lichtenthaler and Wellburn [57]. Briefly, the fresh rice leaf sample (0.2 g) was homogenized in 10 mL of 80% acetone, and the solution was centrifuged at 11,500× *g* for 12 min, and the supernatant was separated in another tube. The absorbance of the collected supernatant was calculated at 663 nm, 645 nm and 470 nm. Eighty percent (80%) acetone was used as blank.

### 4.4. Relative Water Content (RWC) Determination

We used the method of Weatherley [58] to determine the relative water content (RWC). Briefly, the fresh weight (FW) of each sample was measured immediately after harvest. Then, the samples were incubated for 6 h on a shaker in tubes containing 10 mL water at 30 °C. Following the determination of turgid weight (TW), samples were dried at 70 °C for 72 h to determine the dry weight (DW). RWC was calculated as

[(FW − DW)/(TW − DW)] × 100(1)

where fresh weight = FW, turgid weight = TW and dry weight = DW.

### 4.5. Determination of Electrolyte Leakage (%EL)

Electrical leakage (%EL) was measured according to Lutts [59] with some modifications. Briefly, after 15 days in saline solution, 100 mg of leaf tissue were collected from the second youngest leaf of the plants of each pot. Subsequently, the samples were washed three times with deionized water, cut into (10 mm) pieces, placed in 20 mL distilled deionized water, and incubated on a shaker at 30 °C for 6 h. After incubation, electrical conductivity was measured as EC_1_ by an electrical conductivity meter (HI8733, HANNA, USA). The tubes were further incubated in boiling water for 30 min or at 100 °C for 20 min, and the electrical conductivity was measured as EC_2_. Then, the %EL was calculated as

(EC_1_/EC_2_) × 100% (2)

### 4.6. Proline Quantification

We estimated the proline content according to Bates et al. [60] with some modifications. Free proline was extracted from 0.5 g fresh leaf samples using 3% sulfosalicylic acid (10 mL). The 2-mL extraction volume was mixed with 2-mL of a mixture of glacial acetic acid and acid ninhydrin. After being incubated for 1 h at 100 °C, the tubes were placed into ice bath to cool down, 4-mL toluene was added and the upper phase absorbance was measured spectrophotometrically at 520 nm. The free proline was quantified using a standard curve.

### 4.7. Determination of Cellular MDA in Leaves

The status of malondialdehyde (MDA) as an indicator of cellular lipid peroxidation of rice leaves was determined following the method of Heath and Packer [61] and Ali et al. [62]. Briefly, 0.2 g of fresh leaf sample was homogenized in 1.5 mL of 0.1% Trichloroacetic acid. After homogenization, the solution was centrifuged at 11,500× *g* at 4 °C for 15 min, and the supernatant was separated in another tube. In addition, 0.4 mL (400 µL) supernatant was then added in 1 mL of reaction mixture. For blank, 0.4 mL of 0.1% TCA was mixed with 1 mL of reaction mixture. The tube was kept at 95 °C for 30 min in a boiling water bath. After 30 min of boiling, the tube was incubated in an icebox for terminating the reaction, and centrifuged again at 10,000× *g* for 30 min. The absorbance of the collected supernatant was measured at 532 and 600 nm:

$$\begin{array}{c} \text{Calculation of MDA content}\;(\mu \text{mol}\;{\text{g}}^{-1}\text{ FW})\\ =\frac{\left(D532\;-\;D600\right)\;\times \;\mathrm{volume}\;\mathrm{of}\;\mathrm{total}\;\mathrm{mixer}\;\left(0.4\;\mathrm{mL}\;\mathrm{supernatant}\;+\;1\;\mathrm{mL}\;\mathrm{of}\;\mathrm{RM}\right)\;\times \;1000}{\mathrm{Extinction}\;\mathrm{co}\;-\;\mathrm{efficient}\;\left(155{\;\mathrm{mM}}^{-1}\;{\mathrm{cm}}^{-1}\right)\;\times \;\mathrm{sample}\;\mathrm{weight}\;\left(0.2\;g\right)} \end{array}$$

where D532 and D600 denote absorbance reading, and 1000 is used to convert µM from mM of extinction co-efficient.

### 4.8. Determination of Shoot Na^+^ and K^+^

Dried rice shoot was cut into small pieces, and 1 g was digested in 10 mL di-acid mixture of nitric (HNO_3_) and perchloric (HClO_4_) acids (2:1). Then, Na^+^ and K^+^ content were determined by a flame photometer (FP6410, Yuchengtech, China) following the method as described by Krishnamurthy et al. [63].

### 4.9. Statistical Analysis

Data were recorded as means ± standard deviation (SD) from three replications and examined statistically by a three-way analysis of variance (ANOVA). Mean differences were compared through Tukey’s Honestly Significant Difference (HSD) post hoc test at 0.05, 0.01 and 0.001 probability levels, using the statistical package JMP, version 14 from SAS Institute Inc. SigmaPlot, version 14 from Systat Software, Inc., San Jose, CA, USA, (www.systatsoftware.com) was used for principal component analysis (PCA), and Pearson correlation analysis among the variables was performed using program R for windows 4.1.2, R: A language and environment for statistical computing. R Foundation for Statistical Computing, Vienna, Austria. R Core Team (2020). https://www.R-project.org/ (accessed on 4 June 2022).

## 5. Conclusions

The findings of the present study suggested that salinity stress negatively impacted rice growth characteristics like photosynthetic pigments, MDA, proline, electrolyte leakage and Na^+^/K^+^ ratio. In addition, Si pre-treatment significantly improved several important morpho-physiological (shoot height, root length, photosynthetic pigments, electrolyte leakage and relative water content) and cellular biochemical (Proline, MDA, Na^+^/K^+^) parameters particularly in salt-sensitive genotype BRRI dhan48 compared with those in case of Binadhan-10 under salinity. Si is presumably involved with some of the important physiological processes in rice plants under salt stress. Thus, varying levels of Si application might be beneficial in recovery of the growth of salinity-impacted rice plants and thus safeguarding salinity-induced yield loss of rice in the saline-prone areas.

## Data Availability

Not applicable.

## Supplementary Materials

The following supporting information can be downloaded at: https://www.mdpi.com/article/10.3390/plants11141831/s1, Table S1: Three-way analysis of variance (ANOVA) summary table for growth parameters.

## References

[1] Lv B.S., Li X.W., Ma H.Y., Sun Y., Wei L.X., Jiang C.J., Liang Z.W. (2013). Differences in Growth and Physiology of Rice in Response to Different Saline-Alkaline Stress Factors. Agron. J..

[2] Ramayya P.J. (2009). Alkalinity Tolerance in Rice (*Oryza Sativa* L.) Using Molecular Markers (SSRs). J. Adv. Biotechnol..

[3] Shah F., Wu W. (2019). Soil and Crop Management Strategies to Ensure Higher Crop Productivity within Sustainable Environments. Sustainability.

[4] Munns R., Tester M. (2008). Mechanisms of Salinity Tolerance. Annu. Rev. Plant Biol..

[5] Rehman S., Abbas G., Shahid M., Saqib M., Umer Farooq A.B., Hussain M., Murtaza B., Amjad M., Naeem M.A., Farooq A. (2019). Effect of Salinity on Cadmium Tolerance, Ionic Homeostasis and Oxidative Stress Responses in Conocarpus Exposed to Cadmium Stress: Implications for Phytoremediation. Ecotoxicol. Environ. Saf..

[6] Garg N., Bhandari P. (2016). Interactive Effects of Silicon and Arbuscular Mycorrhiza in Modulating Ascorbate-Glutathione Cycle and Antioxidant Scavenging Capacity in Differentially Salt-Tolerant *Cicer Arietinum* L. Genotypes Subjected to Long-Term Salinity. Protoplasma.

[7] Munns R. (2002). Comparative Physiology of Salt and Water Stress. Plant Cell Environ..

[8] Shahid S.A., Zaman M., Heng L. (2018). Soil Salinity: Historical Perspectives and a World Overview of the Problem. Guideline for Salinity Assessment, Mitigation and Adaptation Using Nuclear and Related Techniques.

[9] Sun H., Duan Y., Mitani-Ueno N., Che J., Jia J., Liu J., Guo J., Ma J.F., Gong H. (2020). Tomato Roots Have a Functional Silicon Influx Transporter but Not a Functional Silicon Efflux Transporter. Plant Cell Environ..

[10] Ma J.F. (2004). Role of Silicon in Enhancing the Resistance of Plants to Biotic and Abiotic Stresses. Soil Sci. Plant Nutr..

[11] Ma J.F., Yamaji N. (2006). Silicon Uptake and Accumulation in Higher Plants. Trends Plant Sci..

[12] Saha G., Mostofa M.G., Rahman M.M., Tran L.S.P. (2021). Silicon-Mediated Heat Tolerance in Higher Plants: A Mechanistic Outlook. Plant Physiol. Biochem..

[13] Swain R., Rout G.R. (2020). Silicon Mediated Alleviation of Salinity Stress Regulated by Silicon Transporter Genes (Lsi1 and Lsi2) in Indica Rice. Braz. Arch. Biol. Technol..

[14] Almeida D.M., Margarida Oliveira M., Saibo N.J.M. (2017). Regulation of Na+ and K+ Homeostasis in Plants: Towards Improved Salt Stress Tolerance in Crop Plants. Genet. Mol. Biol..

[15] Yan G., Fan X., Peng M., Yin C., Xiao Z., Liang Y. (2020). Silicon Improves Rice Salinity Resistance by Alleviating Ionic Toxicity and Osmotic Constraint in an Organ-Specific Pattern. Front. Plant Sci..

[16] Gong H.J., Randall D.P., Flowers T.J. (2006). Silicon Deposition in the Root Reduces Sodium Uptake in Rice (*Oryza Sativa* L.) Seedlings by Reducing Bypass Flow. Plant Cell Environ..

[17] Ahmad R., Zaheer S.H., Ismail S. (1992). Role of Silicon in Salt Tolerance of Wheat (*Triticum Aestivum* L.). Plant Sci..

[18] Tuna A.L., Kaya C., Higgs D., Murillo-Amador B., Aydemir S., Girgin A.R. (2008). Silicon Improves Salinity Tolerance in Wheat Plants. Environ. Exp. Bot..

[19] Liang Y., Zhang W., Chen Q., Ding R. (2005). Effects of Silicon on H^+^-ATPase and H^+^-PPase Activity, Fatty Acid Composition and Fluidity of Tonoplast Vesicles from Roots of Salt-Stressed Barley (*Hordeum Vulgare* L.). Environ. Exp. Bot..

[20] Liang Y., Chen Q., Liu Q., Zhang W., Ding R. (2003). Exogenous Silicon (Si) Increases Antioxidant Enzyme Activity and Reduces Lipid Peroxidation in Roots of Salt-Stressed Barley (*Hordeum Vulgare* L.). J. Plant Physiol..

[21] Liang Y., Shen Q., Shen Z., Ma T. (1996). Effects of Silicon on Salinity Tolerance of Two Barley Cultivars. J. Plant Nutr..

[22] Ashraf M., Rahmatullah, Afzal M., Ahmed R., Mujeeb F., Sarwar A., Ali L. (2010). Alleviation of Detrimental Effects of NaCl by Silicon Nutrition in Salt-Sensitive and Salt-Tolerant Genotypes of Sugarcane (*Saccharum Officinarum* L.). Plant Soil.

[23] Ashraf M., Rahmatullah, Ahmad R., Bhatti A.S., Afzal M., Sarwar A., Maqsood M.A., Kanwal S. (2010). Amelioration of Salt Stress in Sugarcane (*Saccharum Officinarum* L.) by Supplying Potassium and Silicon in Hydroponics. Pedosphere.

[24] Lee S.K., Sohn E.Y., Hamayun M., Yoon J.Y., Lee I.J. (2010). Effect of Silicon on Growth and Salinity Stress of Soybean Plant Grown under Hydroponic System. Agrofor. Syst..

[25] Savvas D., Giotis D., Chatzieustratiou E., Bakea M., Patakioutas G. (2009). Silicon Supply in Soilless Cultivations of Zucchini Alleviates Stress Induced by Salinity and Powdery Mildew Infections. Environ. Exp. Bot..

[26] Yan G.C., Nikolic M., Ye M.J., Xiao Z.X., Liang Y.C., Causey G.W. (2018). Cytological Investigations with the Electron Microscope.

[27] Liang X., Fang S., Ji W., Zheng D. (2015). The Positive Effects of Silicon on Rice Seedlings Under Saline-Alkali Mixed Stress. Commun. Soil Sci. Plant Anal..

[28] Ashraf M., Akram N.A., Al-Qurainy F., Foolad M.R. (2011). Drought Tolerance. Roles of Organic Osmolytes, Growth Regulators, and Mineral Nutrients.

[29] Vyrides I., Stuckey D.C. (2017). Compatible Solute Addition to Biological Systems Treating Waste/Wastewater to Counteract Osmotic and Other Environmental Stresses: A Review. Crit. Rev. Biotechnol..

[30] El-Shintinawy F., El-Shourbagy M.N. (2001). Alleviation of Changes in Protein Metabolism in NaCl-Stressed Wheat Seedlings by Thiamine. Biol. Plant..

[31] Saxena M., Saxena J., Nema R., Dharmendra S., Abhishek G. (2013). Phytochemistry of Medicinal Plants. J. Pharmacogn. Phytochem. Phytochem..

[32] Cuin T.A., Shabala S. (2007). Amino Acids Regulate Salinity-Induced Potassium Efflux in Barley Root Epidermis. Planta.

[33] Yan G., Fan X., Zheng W., Gao Z., Yin C., Li T., Liang Y. (2021). Silicon Alleviates Salt Stress-Induced Potassium Deficiency by Promoting Potassium Uptake and Translocation in Rice (*Oryza Sativa* L.). J. Plant Physiol..

[34] Farooq M.A., Saqib Z.A., Akhtar J. (2015). Silicon-Mediated Oxidative Stress Tolerance and Genetic Variability in Rice (*Oryza Sativa* L.) Grown under Combined Stress of Salinity and Boron Toxicity. Turk. J. Agric. For..

[35] Fatikhasari Z., Rachmawati D. (2020). Growth and Oxidative Defense Response to Silicon Application on Rice (*Oryza Sativa* L. ‘Sembada Merah’) under Salinity Stress. AIP Conf. Proc..

[36] Flam-Shepherd R., Huynh W.Q., Coskun D., Hamam A.M., Britto D.T., Kronzucker H.J. (2018). Membrane Fluxes, Bypass Flows, and Sodium Stress in Rice: The Influence of Silicon. J. Exp. Bot..

[37] Hasanuzzaman M., Bhuyan M.H.M.B., Zulfiqar F., Raza A., Mohsin S.M., Al Mahmud J., Fujita M., Fotopoulos V. (2020). Reactive Oxygen Species and Antioxidant Defense in Plants under Abiotic Stress: Revisiting the Crucial Role of a Universal Defense Regulator. Antioxidants.

[38] Wu W., Zhang Q., Ervin E.H., Yang Z., Zhang X. (2017). Physiological Mechanism of Enhancing Salt Stress Tolerance of Perennial Ryegrass by 24-Epibrassinolide. Front. Plant Sci..

[39] Hafez Y., Mazrou Y., Abdelaal K. (2020). Silicon Foliar Application Mitigates Salt Stress in Sweet Pepper Plants by Enhancing Water Status, Photosynthesis, Antioxidant Enzyme Activity and Fruit Yield. Plants.

[40] Matoh T., Kairusmee P., Takahashi E. (1986). Salt-Induced Damage to Rice Plants and Alleviation Effect of Silicate. Soil Sci. Plant Nutr..

[41] Zhu Y.X., Xu X.B., Hu Y.H., Han W.H., Yin J.L., Li H.L., Gong H.J. (2015). Silicon Improves Salt Tolerance by Increasing Root Water Uptake in *Cucumis Sativus* L.. Plant Cell Rep..

[42] Gupta B., Huang B. (2014). Mechanism of Salinity Tolerance in Plants: Physiological, Biochemical, and Molecular Characterization. Int. J. Genom..

[43] Parida A.K., Das A.B. (2005). Salt Tolerance and Salinity Effects on Plants: A Review. Ecotoxicol. Environ. Saf..

[44] Sattar A., Cheema M.A., Abbas T., Sher A., Ijaz M., Hussain M. (2017). Separate and Combined Effects of Silicon and Selenium on Salt Tolerance of Wheat Plants. Russ. J. Plant Physiol..

[45] Yin L., Wang S., Li J., Tanaka K., Oka M. (2013). Application of Silicon Improves Salt Tolerance through Ameliorating Osmotic and Ionic Stresses in the Seedling of Sorghum Bicolor. Acta Physiol. Plant..

[46] Zhu Y.X., Gong H.J., Yin J.L. (2019). Role of Silicon in Mediating Salt Tolerance in Plants: A Review. Plants.

[47] Liang Y. (1999). Effects of Silicon on Enzyme Activity and Sodium, Potassium and Calcium Concentration in Barley under Salt Stress. Plant Soil.

[48] Wang X.S., Han J.G. (2007). Effects of NaCl and Silicon on Ion Distribution in the Roots, Shoots and Leaves of Two Alfalfa Cultivars with Different Salt Tolerance. Soil Sci. Plant Nutr..

[49] Fleck A.T., Nye T., Repenning C., Stahl F., Zahn M., Schenk M.K. (2011). Silicon Enhances Suberization and Lignification in Roots of Rice (*Oryza Sativa*). J. Exp. Bot..

[50] Fleck A.T., Schulze S., Hinrichs M., Specht A., Waßmann F., Schreiber L., Schenk M.K. (2015). Silicon Promotes Exodermal Casparian Band Formation in Si-Accumulating and Si-Excluding Species by Forming Phenol Complexes. PLoS ONE.

[51] Hniličková H., Hnilička F., Orsák M., Hejnák V. (2019). Effect of Salt Stress on Growth, Electrolyte Leakage, Na+ and k+ Content in Selected Plant Species. Plant Soil Environ..

[52] Polash M.A.S., Sakil M.A., Tahjib-Ul-Arif M., Hossain M.A. (2018). Effect of Salinity on Osmolytes and Relative Water Content of Selected Rice Genotypes. Trop. Plant Res..

[53] Ibrahim M.A., Merwad A.M., Elnaka E.A., Burras C.L., Follett L. (2016). Application of Silicon Ameliorated Salinity Stress and Improved Wheat Yield. J. Soil Sci. Environ. Manag..

[54] Rios J.A., de Rodrigues F.Á., Debona D., Silva L.C. (2014). Photosynthetic Gas Exchange in Leaves of Wheat Plants Supplied with Silicon and Infected with Pyricularia Oryzae. Acta Physiol. Plant..

[55] Gregorio G.B., Senadhira D., Mendoza R.D., Division B. (1997). Screening Rice for Salinity Tolerance.

[56] Arnon D.I. (1949). Copper Enzymes in Isolated Chloroplasts. Polyphenoloxidase in Beta Vulgaris; Plant Physiol..

[57] Lichtenthaler H.K., Wellburn A.R. (1983). Determinations of Total Carotenoids and Chlorophylls a and b of Leaf Extracts in Different Solvents. Biochem. Soc. Trans..

[58] Weatherley P.E. (1950). Studies in the Water Relations of the Cotton Plant: I. the Field Measurement of Water Deficits in Leaves. New Phytol..

[59] Lutts S. (1996). NaCl-Induced Senescence in Leaves of Rice (*Oryza Sativa* L.) Cultivars Differing.Pdf. Ann. Bot..

[60] Bates L.S., Waldren R.P., Teare I.D. (1973). Short Communication Rapid Determination of Free Proline for water-stress Studies. Plant Soil.

[61] Heath R.L., Packer L. (1968). Photoperoxidation in Isolated Chloroplasts. I. Kinetics and Stoichiometry of Fatty Acid Peroxidation. Arch. Biochem. Biophys..

[62] Ali M.B., Hahn E.J., Paek K.Y. (2005). Effects of Light Intensities on Antioxidant Enzymes and Malondialdehyde Content during Short-Term Acclimatization on Micropropagated Phalaenopsis Plantlet. Environ. Exp. Bot..

[63] Krishnamurthy S.L., Sharma P.C., Sharma S.K., Batra V., Kumar V., Rao L.V.S. (2016). Effect of Salinity and Use of Stress Indices of Morphological and Physiological Traits at the Seedling Stage in Rice. Indian J. Exp. Biol..

