# Supplementary material for: Assessing Silicon-Mediated Growth Performances in Contrasting Rice Cultivars under Salt Stress
Source: Plants (Basel). 2022 Jul 13;11(14):1831. doi: 10.3390/plants11141831 (PMC9324038; doi:10.3390/plants11141831)
Supplement: Supplementary file 1 [file plants-11-01831-s001.zip › plants-1781462-supplementary.pdf]

Table S1. Three-way analysis of variance (ANOVA) summary table for growth parameters

Variables	Shoot height		Root length		Shoot fresh wt.		Shoot dry wt.		Root fresh wt.		Root dry wt.	
	F	P value	F	P value	F	P value	F	P value	F	P value	F	P value
Variety (V)	55.710	0.0001 ***	10.479	0.004 **	58.693	0.0001 ***	58.801	0.0001 ***	69.001	0.0001 ***	104.242	0.0001 ***
Salinity (S)	28.645	0.0001 ***	1.420	0.245 ns	410.706	0.0001 ***	140.649	0.0001 ***	290.924	0.0001 ***	474.245	0.0001 ***
Treatment (T)	19.086	0.0001 ***	0.376	0.690 ns	2.922	0.073 ns	5.142	0.014 *	10.759	0.001 ***	25.416	0.0001 ***
V×S	28.645	0.0001 ***	6.955	0.014 *	3.374	0.079 ns	0.000	0.995 ns	1.810	0.191 ns	8.824	0.007 **
V×T	0.211	0.812 ns	24.246	0.0001 ***	4.797	0.018 *	3.691	0.040 *	0.567	0.575 ns	3.822	0.036 *
S×T	2.644	0.092 ns	19.442	0.0001 ***	10.067	0.001 ***	1.884	0.174 ns	7.342	0.003 **	29.717	0.0001 ***
V×S×T	15.352	0.0001 ***	13.925	0.0001 ***	2.748	0.084 ns	1.295	0.293 ns	1.724	0.200 ns	7.293	0.003 **

* $P < 0.05$; ** $P < 0.01$ and *** $P < 0.001$; ns, not significant; Variety means two rice cultivars *viz.* BRRI dhan48 and Binadhan-10; Salinity means a saline (at 10 dSM^{-1}) and a non-saline growing conditions; Treatment means three doses of Silicon (Si) pre-treatment *viz.* 0, 1 and 2 mM used in this experiment.
